# Anti-HMGCR myopathy mimicking facioscapulohumeral muscular dystrophy

**DOI:** 10.1515/med-2024-1033

**Published:** 2024-09-04

**Authors:** Andreas Albert Braun, Monika Atiya, Katja Göhner, Tibor Hortobagyi, Tobias Burkhardt, Bettina Schreiner

**Affiliations:** Department of Neurology, University Hospital Zurich, Zurich, Switzerland; Faculty of Medicine, University of Zurich, Zurich, Switzerland; Practice “Neurologie Männedorf”, Männedorf, Switzerland; Department of Rheumatology, University Hospital Zurich, Zurich, Switzerland; Institute of Neuropathology, University Hospital Zurich, Zurich, Switzerland; Practice “Dr. Tobias Burkhardt”, Männedorf, Switzerland

**Keywords:** case report, immune-mediated necrotizing myopathy, facioscapulohumeral muscular dystrophy, FSHD, anti-HMG-CoA reductase antibodies

## Abstract

**Introduction:**

Statin use can lead to various muscle-related issues, including benign creatine kinase (CK) elevations, myalgias, toxic myopathies, rhabdomyolysis, and immune-mediated necrotizing myositis (IMNM), which primarily affects older males. IMNM presents with proximal muscle weakness, elevated CK levels, and specific antibodies.

**Case presentation:**

We describe a 72-year-old patient with muscle weakness persisting for over 3 years after statin therapy. Initially suspected to have a genetic disorder, further testing revealed elevated anti-3-hydroxy-3-methylglutaryl coenzyme A reductase (HMGCR) antibodies, indicating immune-mediated myopathy. Despite the absence of inflammatory changes on biopsy, the patient responded positively to immune therapy.

**Conclusion:**

This case highlights challenges in diagnosing immune-mediated myopathy, especially in older patients with atypical presentations. Testing for HMGCR antibodies can aid in diagnosis, particularly when inflammatory markers are absent. Awareness of red flags, such as delayed symptom onset and response to prednisone, is crucial for accurate diagnosis and management.

## 1 Case report

A 72-year-old male patient had been suffering from increasing painless, bilateral, proximal pareses, pronounced in the arms, with an insidious onset for several years.

In 2012, he suffered a left-hemispheric stroke with a residual right-sided paresis and had been taking atorvastatin ever since. Creatine kinase (CK) was normal in 2012. In 2015, he could still go ibex hunting in the Alps, but a weakness, particularly in the formerly unaffected left arm appeared slowly. In 2021, he could not climb the raised hunting hide due to paresis. He had no problems swallowing, breathing or of ocular muscles. The patient took metformin and dapagliflozin for diabetes, aspirin, and atorvastatin for secondary prophylaxis after stroke and had untreated arterial hypertension. Family history for neuromuscular diseases was negative. He did not consume other drugs.

**Figure 1:**
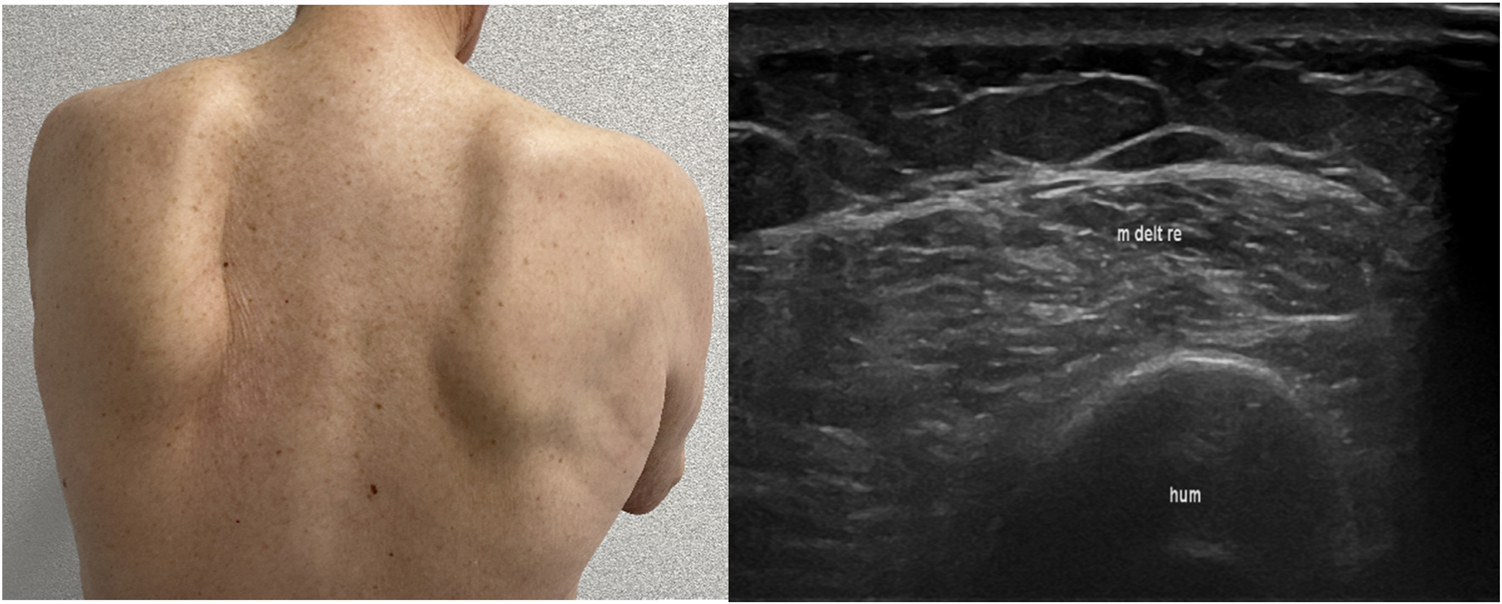
Left image: Photograph showing winged scapula due to shoulder-girdle muscular atrophy, right: muscle sonography, hyperechogenic signal in the deltoid muscle on the right side (m. delt re: right deltoid muscle; hum: humerus bone).

**Figure 2:**
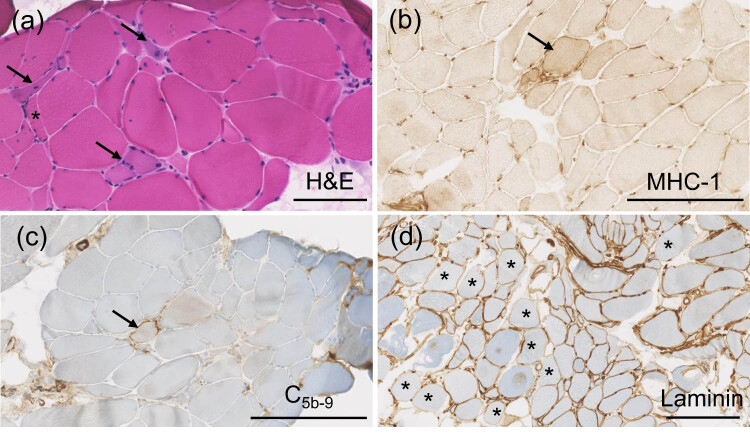
Biopsy of the deltoid muscle, showing non-specific myopathic feature with increased variation of fiber size including small fibers (asterisk), basophilic regenerative fibers (arrows), intracytoplasmic nuclei (a). Immunohistochemistry: HLA-1 (arrow, b) and the membrane attack complex (C_5b-9_) (arrow, c) is upregulated in few fibers (arrow), and some fibers show decreased laminin (LAM-89) expression (asterisks, d). Scale bars: 250 μm.

Neurological examination showed slight facial weakness, bilateral winged scapula, muscle atrophies of the proximal arms, abdominal paresis, proximal and arm-accentuated tetraparesis with minimal muscle strength of M2 in the right biceps and M4 in the proximal legs. While biceps reflex was reduced consistently to the degree of muscle atrophy, reflexes in the legs were normal. Peroneal muscles were not affected. The clinical, sonographic, and pathological findings are shown in Figures 1 and 2.

Laboratory results revealed an elevated CK of 3,864 U/L, aspartate aminotransferase of 155 U/L, and alanine aminotransferase 195 U/L An external neurologist had found myopathic and myotonic discharges in the affected muscles, so that a proximal myotonic myopathy (PROMM) was postulated. His right shoulder joint got a cortisone injection. Thereafter his symptoms improved on both sides for some weeks. Therefore, an external neurologist started a daily oral 125 mg methylprednisone trial for 3 days that improved the patient’s gait, arm elevation and the CK level decreased to 791 U/L. The patient refused a whole-body muscle MRI.

**Figure 3:**
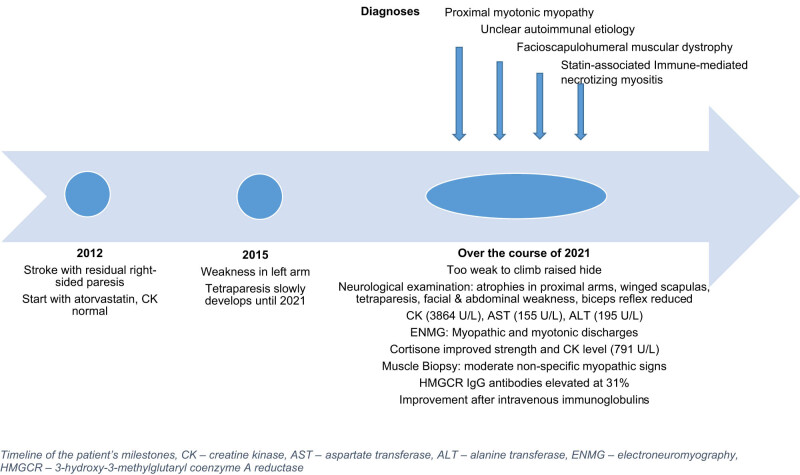
Timeline of the patient’s milestones, CK: creatine kinase; AST: aspartate transferase; ALT: alanine transferase; ENMG: electroneuromyography; HMGCR: 3-hydroxy-3-methylglutaryl coenzyme A reductase.

A biopsy of the deltoid muscle more than a month after the last cortisone treatment showed moderate non-specific myopathic signs. There was increased variation in muscle fiber size and mildly elevated number in internalized nuclei, occasional necrotic and basophilic regenerative as well as small-elongated atrophic fibers without grouping, split fibers, and an absence of inflammatory cell infiltrates (Figure 2a). In few abnormal fibers, sarcolemmal upregulation of MHC-1 (Figure 2b) and of membrane attack complex (C_5b-9_) (Figure 2c) was noted. The expressions of sarcolemmal and sarcolemma-associated proteins were normal, except for lower expression of laminin with antibody LAM-89 in few fibers (Figure 2d). Figure 3 summarizes the clinical timeline.

In summary, we initially suspected a hereditary cause such as facioscapulohumeral muscular dystrophy (FSHD) or limb-girdle muscular dystrophy (LGMD) because of insidious onset, the phenotype of the scapulohumeral muscle atrophy despite only slight facial affection and sparing of the peroneal muscles and non-inflammatory muscle biopsy findings. The less common LGMD (e.g., type 3) would also have fitted the clinical phenotype and the recessive inheritance of some types would have explained the negative family history. Therefore, analysis for LGMD would have been the next diagnostic step in the genetic work-up if we had not decided with the rheumatologists to test for 3-hydroxy-3-methylglutaryl coenzyme A reductase ((HMGCR) IgG antibodies because the CK levels above 1,500 U/L, the biopsy findings and the response to steroids which are more suggestive of a non-genetic diagnosis [1]. In fact, 55% of myopathies with elevated CK referred to rheumatologists finally emerge as inflammatory myopathies [2]. HMGCR IgG antibodies were elevated at 31% (cut-off >5%). Therefore, we finally diagnosed a statin-associated immune-mediated myopathy. The patient improved within few months under the treatment with intravenous immunoglobulins and was able to climb stairs again.


**Ethical approval:** This retrospective review of patient data did not require ethical approval in accordance with local guidelines (Kantonale Ethikkommission Zürich).
**Informed consent:** Informed written consent for publication was obtained from the patient, including the publication of medical images.

## 2 Discussion

Statin-associated immune-mediated necrotizing myopathy or myositis (IMNM) occurs in approximately 1 patient per 100,000 person years [3] with a mean latency ranging from months to 9 years after start of statin use and a broad range of clinical manifestations [4]. In seropositive IMNM, HMGCR antibodies or signal recognition particle antibodies are detected. Anti-HMGCR autoantibodies target the rate-limiting enzyme of cholesterol synthesis, HMG-CoA reductase, leading to activation of the classical complement pathway, resulting in muscle necrosis. This triggers macrophage activation and an inflammatory response, exacerbating muscle injury and impairing muscle regeneration by disrupting myoblast differentiation and contributing to muscle atrophy. Additionally, statins may directly induce apoptosis via mitochondrial dysfunction and oxidative stress, further contributing to muscle cell death. The key pathological features of IMNM are apoptosis, vacuolation, and necrosis [5,6]. MHC class I is increased in areas of active muscle fiber degeneration [7]. Muscle biopsies may show no inflammatory changes due to sampling errors (multifocal inflammation) or after pretreatment with prednisone which can be misleading [8]. Electroneuromyography studies show relatively pronounced myotonic discharges [9] besides myopathic findings. Therefore, it has to be kept in mind that myotonic discharges are not exclusively found in myotonic diseases such as PROMM, but can occur among others in inflammatory myopathies and drug-induced myopathies [10]. They are described in immune-mediated myopathies with and without statin intake [9].

This case report is in line with former studies who showed that 60 or 33% of patients initially presumed to have a FSHD or LGMD actually had antibodies against HGMCR and therefore a treatable condition [7]. Of note, mutations in the HMGCR-gene can lead to a myopathy resembling LGMD. FSHD is a relatively common autosomal-dominant neuromuscular disease with a prevalence of 5–12 per 100,000. It has no full penetrance and 10–30% are *de-novo* mutations. Disease onset is typically between 15 and 30 years, but can be much later [11]. Patient with genetically proven muscular dystrophy such as FSHD usually do not have anti-HMGCR antibodies even though rare cases with two diseases have been reported [12].

Taken together, IMNM with HMGCR should be considered in any proximal myopathy, particularly above 50 years of age. It can appear in the absence of statin intake. Our case is worthy of note due to long latency between statin intake and symptoms, slow progression, myotonic discharges, phenotype of shoulder girdle weakness resembling FSHD/LGMD and absence of inflammatory infiltrates on muscle biopsy. Subacute onset or posterior thigh involvement for the distinction between HMGCR myopathy and FSHD or LGMD were not present [13]. The correct diagnosis is highly relevant, as IMNM responds to treatment with prednisone, methotrexate, intravenous immunoglobulins, or rituximab [14]. Steroids, immunoglobulins, or methotrexate are the first-line treatment with consecutive steroid tapering [15]. As an association of HMGCR myopathy with malignancy has been reported, a cancer screening is recommended [16].
